# Ischemic Heart Disease in Workers at Mayak PA: Latency of Incidence Risk after Radiation Exposure

**DOI:** 10.1371/journal.pone.0096309

**Published:** 2014-05-14

**Authors:** Cristoforo Simonetto, Tamara V. Azizova, Evgenia S. Grigoryeva, Jan C. Kaiser, Helmut Schöllnberger, Markus Eidemüller

**Affiliations:** 1 Helmholtz Zentrum München, Department of Radiation Sciences, Neuherberg, Germany; 2 Southern Urals Biophysics Institute (SUBI), Ozyorsk, Chelyabinsk Region, Russia; Kagoshima University Graduate School of Medical and Dental Sciences, Japan

## Abstract

We present an updated analysis of incidence and mortality from atherosclerotic induced ischemic heart diseases in the cohort of workers at the Mayak Production Association (PA). This cohort constitutes one of the most important sources for the assessment of radiation risk. It is exceptional because it comprises information on several other risk factors. While most of the workers have been exposed to external gamma radiation, a large proportion has additionally been exposed to internal radiation from inhaled plutonium. Compared to a previous study by Azizova et al. 2012, the updated dosimetry system MWDS-2008 has been applied and methods of analysis have been revised. We extend the analysis of the significant incidence risk and observe that main detrimental effects of external radiation exposure occur after more than about 30 years. For mortality, significant risk was found in males with an excess relative risk per dose of 0.09 (95% CI: 0.02; 0.16) 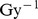 while risk was insignificant for females. With respect to internal radiation exposure no association to risk could be established.

## Introduction

Damage to the heart from high doses of ionizing radiation has been detected in radiotherapy patients [1], [2]. Evidence for an association of lower doses of radiation with the risk of heart diseases was found in the atomic bomb survivors [3] and triggered investigations into several other cohorts. Still, the precise risk for cardiovascular diseases from low dose radiation as well as the related main biological mechanisms remain unknown, although atherosclerosis seems to play a major role [4]. Little [5] summarized the epidemiological evidence for a causal association between moderate- and low-level radiation exposure and circulatory diseases. The meta-analysis [6] supports an association between low doses and low dose rates of ionizing radiation and an excess risk of ischemic heart diseases (IHD). It is interesting to note that within this meta-analysis the Mayak Workers Cohort [7] had the strongest effect on the calculated radiation risk.

The Mayak Production Association (PA) was the first facility in the former Soviet Union for the production of weapon grade plutonium. It comprises several nuclear reactors and a nuclear fuel reprocessing facility [8]. The Mayak workers were exposed to low and medium doses at low dose rates. This together with the fact that these individuals did not have the threatening and traumatic experience of being exposed to the detonation of a nuclear bomb makes this data set especially valuable for cancer- and non-cancer-related risk estimations of general populations. The Mayak Workers Cohort has been the subject of several studies to investigate whether radiation may influence the pathogenesis of circulatory diseases (e.g. [7], [9]–[13]). While early analyses based solely on mortality did not find any effect [9], follow-up studies showed detrimental effects in incidence [7], [10]. In the study by Azizova et al. [12] IHD was analyzed for various lag-times assuming a linear relationship between absorbed dose and risk.

However, while the assumption of a linear relationship may be reasonable for the proof of an association, it is the next logical step to extend this analysis. One of the most important questions relates to the shape of the dose-response at low doses, i.e. whether risk at low doses is larger or smaller compared to the linear interpolation or whether even protective effects may occur [14]. In this respect, the present study adds to the ongoing discussion [6], [15]–[19]. In addition to linear responses more flexible shapes have been applied to consider possible imprints of complex biological effects, which have been observed at low doses in vivo and in vitro (e.g. [20]–[26]). Moreover, our study also comprises a detailed analysis of the time progression of IHD by applying various age- and exposure-related dose-effect modifications in addition to the classical lag-time approach. Furthermore, we compare the excess relative risk (ERR) models to an excess absolute risk (EAR) model. Another difference to the study by Azizova et al. [12] is the use of updated data that relate to the latest dosimetric evaluations, the so-called Mayak Worker Dosimetry System 2008 (MWDS-2008) [27].

## Materials and Methods

### Materials: Cohort definition

This section summarizes briefly the main characteristics of the cohort, some issues important for the analysis and the differences to previous analyses [7], [12]. For more details on data collection and cohort definition the reader is referred to [28], [29].

#### Study cohort

The study cohort is based on the nuclear workers at Mayak PA, which was founded in 1948. If employed before 1973, workers of the main facilities (reactors, radiochemical and plutonium plants) have been included from the first day of occupation. Persons with acute radiation syndrome have been excluded (43) as well as persons with peculiar variations in the measured internal exposure (57) as the variations may be indicative for unusual uptake scenarios.

The cohort includes 18,797 workers of which 4,741 are women. The total number of persons has slightly changed compared to earlier analyses due to revision of occupational and clinical data. As information on internal radiation burden is not available for everybody, analysis on internal doses is restricted to 9,689 workers. This includes 4,162 workers at the reactors for which internal doses are unknown but are assumed to be vanishing. Table 1 summarizes the number of persons, person years and cases on which the different analyses performed in this work are based. The difference in numbers comparing incidence to mortality arises from different follow up as will be explained below. Mean age of first employment was 25 (5% and 95% percentiles: 17; 45) years. First IHD incidence occurred at a mean age of 56 (41; 75) years, while workers dropped out of the incidence cohort at a mean age of 49 (21; 75) years. The average age of IHD mortality is 64 (42; 84) years and is close to the mean age for leaving the mortality cohort of 63 (24; 84) years. At the end of follow up, the mean age of workers in the mortality cohort is 70 years.

**Table 1:** Number of persons, person years and cases for the different analyses performed in this work, separately for males and females.

		External doses		Internal doses	
		Incidence	Mortality	Incidence	Mortality
Persons	M	10,155	14,056	4,843	7,075
	F	3,176	4,741	1,692	2,614
Person years	M	201,320	516,145	76,033	181,784
	F	78,727	203,087	28,199	67,653
IHD Cases	M	3,888	2,083	2,196	1,107
	F	1,721	469	893	246

#### Endpoints

Diagnostic methods for IHD have improved over time. For this study, however, verification was based for all cases on clinical symptoms and signs as well as electrocardiogram readings. Ischemic heart diseases are specified by the ICD-9 codes 410–414. For incidence 98% of the cases refer to ICD-9 code 414 for both males and females. The vast majority is attributed to ICD-9 code 414.0, coronary atherosclerosis. This dominance can be understood considering that coronary atherosclerosis is typically the first in a chain of cardiovascular complications.

For most of the deaths in Ozyorsk, full clinical documentation is available. In order to focus on atherosclerotic induced ischemic heart diseases, deaths from Ozyorsk residents have been considered only if the underlying disease was coded as 414.0. This specification excluded less than 2% of all IHD mortality cases.

#### Radiation exposure and dosimetry

Yearly dose estimates from external radiation sources have been assigned to each worker. This assignment is based on film badge readings. Nevertheless, there remain uncertainties, for example due to the fact that the film badge response depends on energy and angle of the penetrating radiation [30]. In this work, we apply the Mayak Worker Dosimetry System 2008. For more information on the dosimetry system, the reader is referred to [30]. The mean total dose in male workers is 0.62 (5% and 95% percentiles: 0.0; 2.8) Gy and 0.51 (0.0; 2.4) Gy in female workers. Most persons received major exposure during the first years of employment as health and safety measures improved over time. This results in an average of the median ages at exposure of 29 (19; 49) years. The mean duration of half exposure is 5 (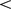
1; 19) years. Restricting to certain dose groups, the mean duration of half exposure is 3 years (for workers with total dose below 0.1 Gy), 6 years (above 0.1 Gy but below 1 Gy) and 4 years (above 1 Gy).

Plutonium-239 body burden was evaluated from results of biophysical examinations and autopsy data [27], [30]. Yearly dose estimates to several organs have been derived. However, there is no specific estimate for the dose to the heart. Thus, dose to liver is used as a surrogate in this work. Measurements of internal radiation burden have been conducted only for 29% of the workers. Within these persons an average total internal dose of 0.27 (5% and 95% percentiles: 0.006; 2.0) Gy is found for males and 0.48 (0.008; 5.0) Gy for females. Plutonium-239 is degraded only slowly in the body. Thus, internal radiation burden is lifelong. Finally, uncertainty in internal doses is rather large [31].

#### Follow up

**Figure 1:**
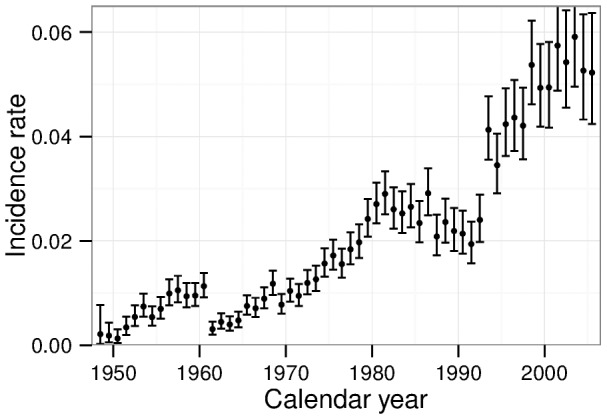
Crude IHD incidence rates of Mayak workers and 95% confidence intervals for different calendar years. The crude rate is defined by the number of cases per person years.

In fig. 1 the number of incident cases per person years is plotted for different calendar years. Apart from the overall increase due to aging of the cohort some steps and kinks are evident. The relatively strongest step emerges after 1960 with a more than three-fold decrease. The underlying reasons for this step are not known to the authors. Possibly, medical examinations have become substantially more accurate during the 50 s such that prevalent cases could be detected in addition to recently developed cases. In any case, this step is very likely not due to radiation. However, it might impede proper analysis of radiation induced risk and its time dependence. Thus, all person years until end of 1960 are excluded from the incidence analysis.

The step in early 90 s very likely is connected to the transition of the Soviet Union to Russia, which came along with individual stress and major changes of the health care system. In this period, also changes in the mortality data are evident and are known to exist in the general population as well [32]. We model the hazard with a non-differentiable calendar-year dependence, cf. section A.2 in Appendix S1.

Another complication arises for the investigation of risk from internal doses. Biophysical examinations for determination of internal radiation burden have often been carried out decades after first employment. In particular, low radiation burdens have been determined mainly in recent decades. This poses a selection of persons: Only workers that became sufficiently old may have been examined for internal radiation. Hence workers with measured low internal doses have a longer life on average – as a prerequisite of the measurement and not as a consequence of the low dose. To overcome this problem, person years before the date of first biophysical examination are excluded in the analyses of internal doses. To assure that examinations have not been performed in the context of a recently developed disease, an additional year is excluded after the first biophysical examination.

In summary, the begin of follow up for each person is given by the latest of: date of first employment, January 1, 1961 for the incidence analysis or one year after the date of first biophysical measurement when studying effects of internal radiation. The end of follow up is defined by the earliest of: December 31, 2005, date of first IHD incidence/death or date of last information. Date of last information refers to the availability of clinical data in Ozyorsk. An exception is mortality among migrants for which information after this date is provided by the Southern Urals Biophysics Institute (SUBI) Laboratory of Epidemiology. However, losses of follow up occur more frequently after emigration from Ozyorsk [12].

#### Ethics Statement

This record-based epidemiological study did not require any contact with the cohort members. The project was reviewed and approved by the Institutional Review Board of the Southern Urals Biophysics Institute (SUBI).

### Methods: Statistical analysis

#### Baseline data

One of the strengths of the Mayak Workers Cohort is the comparatively large number of covariables that can be accounted for. In addition to the information on birth date and date of employment, there is also information on smoking (non-smoker, smoker and ex-smoker, unknown) and drinking status (non-drinker, drinker and ex-drinker, unknown), on body mass index (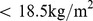, normal, 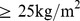, unknown), on blood pressure (normal, above 140/90 mmHg, unknown) and on work plant (work only in reactor, only in radiochemical plant or reactor, at least for some time in plutonium plant). To factor out the impact of radiation on body mass index and blood pressure, information from the pre-employment medical examination is used. On the other hand, all available information until end of follow up was used for smoking and drinking status. This information is based on interviews at several medical examinations. Non-smokers/non-drinkers are workers who always have claimed never to have been a smoker/drinker. More details on the collection of information on risk factors can be found in [28]. Finally, there is also information on the date of emigration. A different hazard can be expected after emigration due to another lifestyle but also due to different quality of follow up: For Ozyorsk residents full clinical data are available while for emigrants information relies on death certificates. Moreover, since several years, information on vital status and cause of death of emigrants can be assessed via mail contact only.

All information has been implemented in a continuous function given in section A.2 in Appendix S1. For each analysis, only parameters significantly deviating from zero are included.

#### Time dependence of risk of external doses

**Figure 2:**
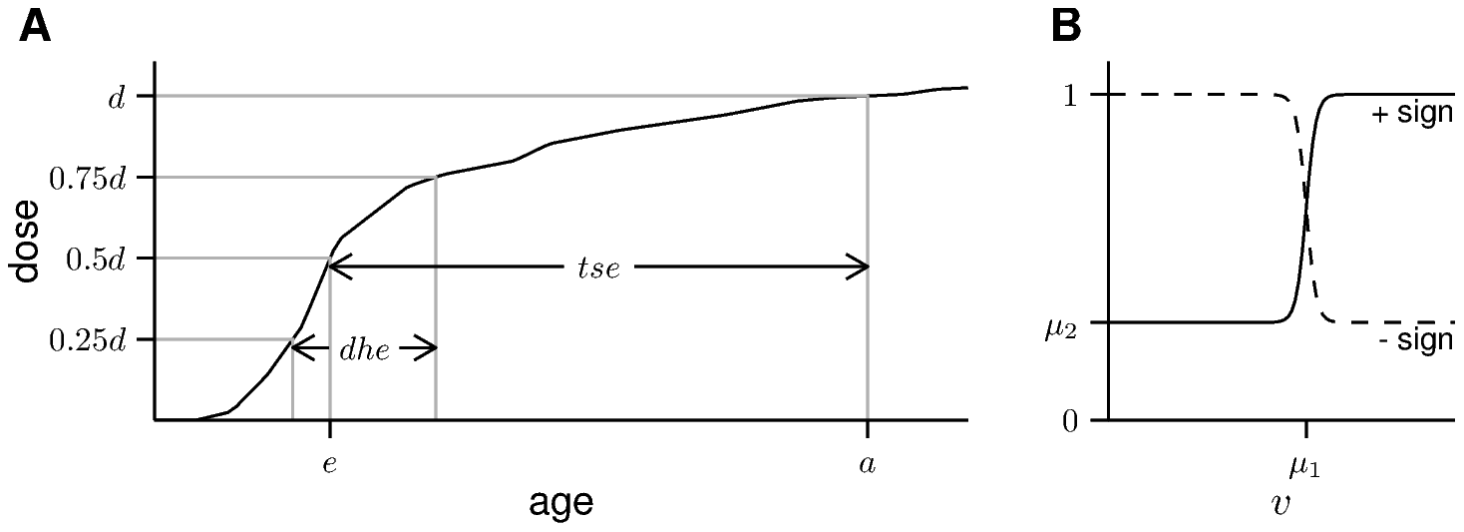
Illustrations of the variables and functions used for dose modification. (A) Definition of the time variables. (B) The step function in eq. (2).

The time dependency of the risk of external radiation is examined within the linear no-threshold (LNT) model. Different time scales might influence progression of the disease. To disentangle the relevant ones we independently test modification of the dose response with the time variables age attained (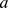), age at median exposure (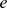), time since median exposure (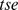) and duration of half exposure (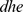). Age at median exposure is defined to be the age at which half of the hitherto accumulated dose was received, time since median exposure is 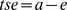. In order to suppress years with minor exposures, we apply 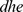 instead of the duration of the whole exposure. It is defined to be the time span from end of the first to beginning of the last quartile of the hitherto accumulated dose. An illustration of these definitions is shown in fig. 2A.

For each time variable, we test modification with two different functions. The first is exponential in time:
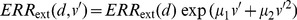
(1)where 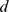 refers to dose, 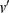 may be any of 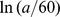, 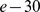, 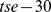 and 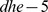 and all time variables are given in units of years. Secondly we test with the logistic function, a (smoothed) step function:
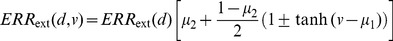
(2)being 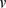 any of 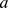, 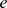, 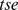 and 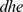. The step function jumps between two levels in the region around 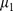, cf. fig. 2B. Instead of an instantaneous jump we use a function with finite slope. This is motivated by the fact that doses are given only as yearly averages and, moreover, instantaneous steps in time also do not seem to be biologically plausible. A technical discussion against the use of instantaneous steps when comparing models by their deviance can be found in section A.3 in Appendix S1.

Both functions are tested for 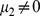 only in case of significant 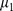. The signs in the step function are chosen such that for 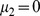 the step function yields virtually no risk for young ages, short times since exposure, old ages at exposure and long durations of exposure. Thus, the 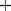 sign in the step function is applied to age and 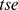, while the 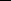 sign is applied to age at median exposure and 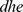.

If several dose modifications improve the fit at a p-value of 0.05 in the 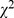-distribution, the Akaike information Criterion (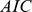) [33], [34] is applied for a ranking. It is given by the sum of the deviance and twice the number of parameters.

Finally, it should be noted that in the section on time dependence of risk we do not employ time-lagged doses, i.e. doses accumulated over a certain period in the past. Time-lagging is often performed to exclude exposures obtained so recently that a connection to the disease seems unlikely. Instead, we prefer to use the total, hitherto accumulated dose but modify the risk with time since median exposure as explained above. In general, both approaches have their pros and cons in the description of cardiovascular diseases. Applying a simple lag-time does not allow to model for any time dependence but a step. In more generalized models such as in the BEIR VI report [35] it is implicitly assumed that each exposure adds independently to the risk, which may not be true for IHD. On the other hand, in applying modification with time since median exposure, differences in the exposure history are completely ignored as long as the total dose is fixed.

However, we want to compare modification by 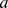, 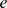, 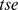 and 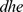. This is possible only within the framework of dose-response modification (cf. eqs. (1) and (2)) and not within the concept of time-lagging. Moreover, employing modification by time since median exposure permits to focus on late effects even when there is also an earlier effect. In contrast, the concept of a minimal lag-time fails in this case as can be illustrated with the following example. For male incidence there are 5288 person years in the dose range [0.1 Gy, 0.2 Gy] when using a lag-time of 30 years. Of these, 85% belong to persons with total doses of more than 0.2 Gy and 28% to persons with even more than 0.5 Gy. When using a lag-time of 30 years, these persons contribute to the risk estimate for the dose range [0.1 Gy, 0.2 Gy]. However, when there is an early effect, the risk of these persons may be affected substantially by the exposures obtained within the lag-time thus introducing a bias to the the risk estimate at low doses. In contrast, when employing modification by time since median exposure, the model for the risk is always based on the (hitherto accumulated) total dose.

## Results

In the following we will first assess the average risk in an LNT framework. Next we will study modification of risk by time and other risk factors. Finally, the shape of the dose-response relationship will be analyzed.

### Analysis with linear dose response and without effect modification

#### External dose

Results of the analysis without any risk modification are shown in table 2 for various lag-times. For male incidence we observe external doses being significantly associated with risk, irrespective of the lag-time. The best fit is found for a lag-time of 30 years, with a substantially higher excess relative risk compared to shorter lag-times. For females, risk is not significant for lag-times shorter than 30 years. But again, the best fit is found for a lag-time of 30 years, with a substantially higher excess relative risk. The lag-time of 30 years is selected with very high significance. For mortality, we observe borderline significant risk for males. It remains significant, if restricting the follow up to the time in Ozyorsk, except for the analysis with a lag-time of 30 years. A substantial difference for the different lag-times cannot be observed. Female risk estimates do not show any deviation from zero but are based on a much smaller number of cases.

**Table 2:** Excess relative risk per dose for external gamma radiation for various lag-times.

		0 years lag-time		10 years lag-time		20 years lag-time		30 years lag-time	
		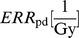	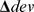	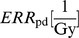	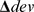	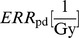	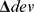	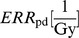	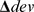
Inc.	M	0.10	1.9	0.11	0.0	0.10	4.9	0.20	−4.9
		(0.04;0.18)		(0.05;0.19)		(0.03;0.18)		(0.10;0.32)	
	F	0.04	0.4	0.05	0.0	0.07	−1.3	0.29	−19.5
		(−0.04;0.14)		(−0.04;0.15)		(−0.02;0.18)		(0.15;0.47)	
Mort.	M	0.08	0.8	0.09	0.0	0.09	−0.8	0.07	3.7
		(0.02;0.15)		(0.02;0.16)		(0.03;0.17)		(0.00;0.15)	
	F	−0.01	0.0	0.00	0.0	0.00	0.0	0.01	0.0
		(−0.13;0.14)		(−0.12;0.15)		(−0.12;0.16)		(−0.12;0.18)	

Excess relative risk per dose (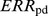) with 95% confidence levels. The LNT model was applied using various lag-times. Deviances (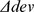) are compared to the analysis with a lag-time of ten years.

**Figure 3:**
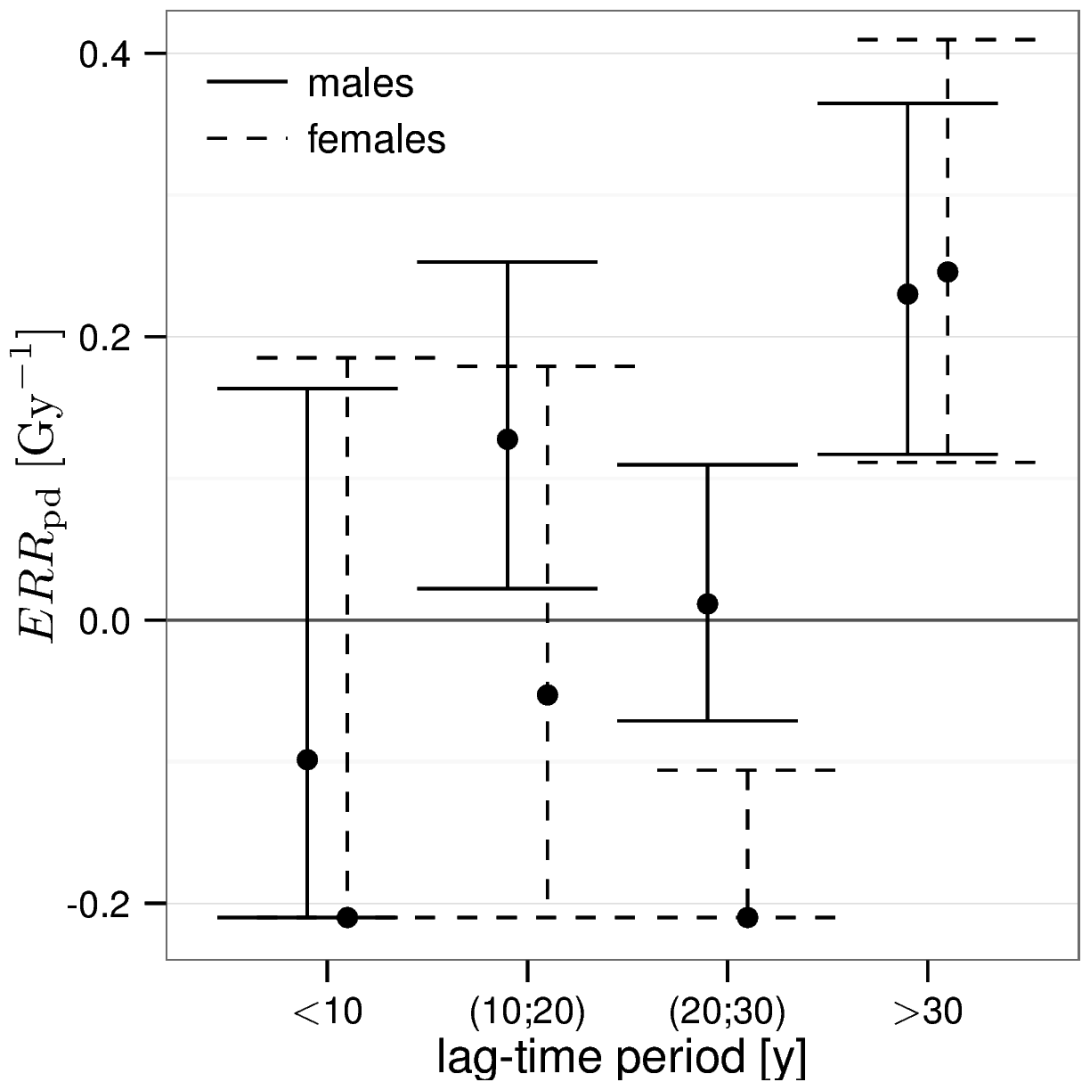
Excess relative risk per dose of external gamma radiation and 95% confidence intervals for incidence, separately for different lag-time periods. Risk has been assigned to the lag-time as in the BEIR VI report [35]. The 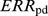 is bound from below by the requirement of a positive hazard for the workers with highest doses.

The strong dependence of incidence on the lag-time deserves a closer look. In fig. 3, we show the result of a fit that is categorical in the lag-time. The fit has been performed analogous to the model of the BEIR VI report [35]. That is, the ERR of an individual is obtained by adding the risks from single exposures. Here, the 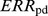 associated to a single exposure depends on the time elapsed since this exposure. The increase in 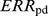 from exposures that are less than 30 years ago to exposures more than 30 years ago is evident. In addition, for the lag-time period of more than 20 but less than 30 years, a temporary protective effect can be observed for females but not for males.

The analyses of this section were performed without taking into account possible risk modification due to aging of the cohort. To disentangle the time for the development of the disease from other relevant time scales, a more sophisticated analysis will be presented below.

#### Internal dose

To gain statistical power a joint dose response for males and females is assumed for the analysis of internal doses although two baselines are applied – one for each gender. Nevertheless, significant association of risk with internal dose could not be found for either incidence nor mortality. The results can be found in Table S1. Due to absence of a significant effect, we will not pursue the analysis of internal doses. It should, however, be noticed that the estimated excess relative risk increases when disregarding workers with doses of more than a few Gray.

### Time dependence of risk of external doses

We compare impact of the time variables age, age at median exposure, time since median exposure and duration of half exposure. For each time variable, we test modification of the ERR both with an exponential and with a step function according to eqs. (1) and (2), assuming an LNT dose-response relationship.

We find for male and female incidence, a step in time since median exposure to yield the best improvement in deviance. For males, it is the only significant modification found with 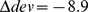 compared to a model without modification and no lag-time. The step is at 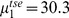 (95% confidence interval: 28.6; 32.0) years and 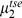 is consistent with zero. For females, several time modifications are significant. However, the best 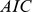 is obtained for a step in 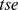 at 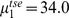 (32.2; 34.9) years, with 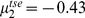 (−1.00; −0.14) and a deviance difference of 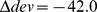. This value for 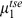 is essentially unaffected when setting 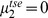, resulting in 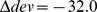. Note that negative 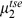 implies a temporary protective effect. The 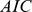 is better by 5.0 points compared to any of the other tested modifications. Therefore, for both genders, time since median exposure governs incidence risk modification and more than 30 years need to elapse before the main detrimental health effects appear.

To confirm this result, we have conducted two statistical tests: First, we amended the baseline function by a step function of time since first employment. As for most persons major exposure was in the first years of employment, time since first employment is strongly related to 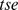. We find that amendment of time since first employment did not considerably change the impact of time since median exposure. This confirms that the 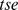 effect is indeed related to dose. Secondly, motivated by the strong changes in the baseline, cf. fig. 1, we repeated the analyses but dropped the calendar years 1992 to 1994. Again, the effect of 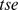 was not changed considerably.

Finally, we repeated the analysis of male and female incidence with an EAR model; for a definition see eq. (A5) in Appendix S1. Again, a step in time since median exposure yields the best improvement in deviance. The position of the step is compatible to the ERR analysis for both male and female incidence. For males the deviance of the EAR model is higher by 1.7 points compared to the ERR model. For females, it is higher even by 14.8 points with 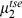 not significantly deviating from zero. Thus – at least within the time modified LNT – an excess relative risk model yields a better description of the data. The existence of the step in 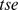, however, is independent of whether an ERR or an EAR model is assumed.

Such a step after a certain time since exposure may be indicative for possibly different mechanisms responsible for the risk before and after the step. Below, we want to focus on the late period for which the main detrimental effects are observed. This can be accomplished by investigating for further effect modification only doses with a time since median exposure of more than 30 years for males (35 years for females). Thereby the period of the negative excess risk is excluded from further investigation. The separation of exposure histories into recent and former exposures replaces the modification by 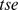. Focusing in this way on the late period only, we repeat the tests for modification. However, we do not find any additional significant modification with any time variable.

In contrast to the incidence analysis, for male mortality none of the tested modifiers pose a significant improvement in the deviance. Repeating the analysis with an EAR model, a step at an age of 65 years is significant but does not pose an improvement in deviance compared to an ERR model without modification. Female mortality has not been tested for modification of the dose response as the dose response itself is not significant, cf. table 2.

### Modification by other risk factors

After having studied modification with time, next we analyze possible interactions between radiation and other risk factors. For this purpose we modify the dose response 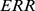 by a factor 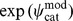 and test the attributes of smoking, drinking, body mass index and blood pressure. To ensure separation of baseline and radiation risk modification of a risk factor, the corresponding variable in the baseline template is also released even if it had previously been shown not to be a significant covariable.

Interestingly, we find significant interaction between radiation and underweight in the male incidence analysis: the 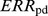 of the late detrimental effect is found to be 7 (1.2; 21) fold increased for persons with a body mass index below 18.5 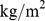. Consistently, also underweight females show increased 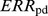 although significance is marginally missed. Moreover, overweight female workers show a marginally non-significant decrease in 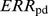. All results for the incidence analysis can be found in Table S2. As the mortality analysis does not have enough power to add sensible information, it is not shown.

### The dose-response relationship of external doses

To determine the most likely dose-response we apply a categorical model as well as twelve different functions that can be found in eq. (A6) and are sketched in fig. A1 in Appendix S1. The goodness of fit is assessed by a series of likelihood-ratio tests. This is possible only between nested models. We have selected a set of functions of which many are nested with each other but typically the likelihood-ratio test cannot decide between the branch of models nested with the LNT and the branch nested with the step model, cf. fig. A2 in Appendix S1. Starting from the models with fewest parameters, comparison is always performed to the currently best model in a series of nested models.

**Figure 4:**
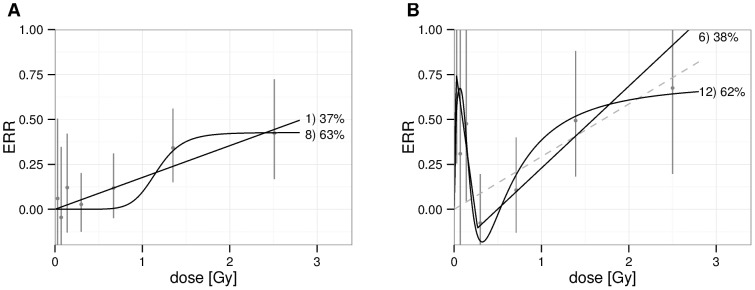
Shape of the dose response in the incidence analysis. (A) Males, time since median exposure 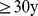. (B) Females, time since median exposure 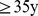. Vertical bars indicate 95% confidence intervals of the categorical analysis. As black solid lines, we present models that pass the likelihood-ratio test. They are annotated by a reference number (cf. fig. A1 in Appendix S1) and their Akaike weight, which is proportional to 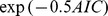. Models that contribute an Akaike weight of less than 5% are not shown. In the right panel, the LNT model is presented as a reference.

As explained in previous sections, we observe two temporarily separated radiation effects in incidence. While the late effect turned out to be more relevant for the goodness of fit, inclusion of the earlier, temporary protective effect was also highly significant for females. Therefore, we perform two different analyses on incidence risk: first, applying the different models of the dose-response relationship only to the late, main detrimental effect (fig. 4) and secondly a fit without restriction on time since median exposure (not shown), thus averaging over the full follow up time. As mortality risk turned out to be non-significant for females, the mortality dose-response relationship is investigated only for males (not shown). The mortality analysis is performed without restriction on time since median exposure.

First, we present the results for male incidence with restriction on time since exposure as it is the analysis with the largest number of cases and because the restriction yields a better fit. As the best model we find a step at 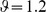 (0.7; 1.5) Gy to an excess relative risk of 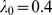 (0.2; 0.7), cf. fig. 4A. Its 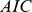 is lower by 1.1 points compared to an LNT model with a slope of 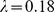 (0.09; 0.27) 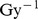. However, the LNT model passes the likelihood ratio test, too.

Secondly, we note the significant excess in risk for doses below 200 mGy, not only for female incidence, fig. 4B, but also for any of the analyses not shown. Indeed, in any of the above mentioned analyses, either model 6) or 12) yields the best fit as these models are able to describe the peak. However, as will be discussed in the next section, this peak might well be induced by confounding. As the peak has proven to impact the choice of models, the validity of the selected models may be partially limited also at higher doses. Hence, we present in table 3 only the results of the categorical analysis.

**Table 3:** Excess relative risk by categories of cumulative external doses.

Dose [Gy]	Incidence		Incidence		Mortality
	(restriction on 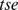 )		(no restriction)		(no restriction)
	M	F	M	F	M
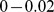	0	0	0	0	0
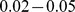	0.06(-0.28;0.50)	1.19(0.25;2.55)	0.16(−0.05;0.42)	0.13(−0.11;0.41)	0.11(−0.12;0.40)
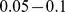	−0.05(−0.35;0.35)	0.31(−0.25;1.12)	0.05(−0.12;0.27)	0.20(−0.03;0.47)	0.05(−0.15;0.31)
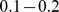	0.12(−0.13;0.42)	0.48(0.05;1.03)	0.23(0.05;0.45)	0.09(−0.10;0.32)	0.08(−0.12;0.31)
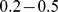	0.03(−0.12;0.20)	−0.08(−0.29;0.20)	0.11(−0.05;0.29)	−0.03(−0.20;0.18)	−0.09(−0.24;0.09)
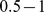	0.12(−0.05;0.31)	0.11(−0.13;0.40)	0.21(0.03;0.41)	−0.08(−0.26;0.13)	0.02(−0.16;0.24)
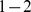	0.34(0.15;0.56)	0.49(0.18;0.88)	0.29(0.10;0.53)	0.02(−0.18;0.27)	0.11(−0.09;0.35)
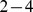	0.42(0.17;0.72)	0.67(0.20;1.30)	0.44(0.20;0.74)	0.13(−0.14;0.48)	0.34(0.08;0.67)
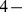	0.40(−0.38;1.7)	3.02(−0.37;11.9)	0.22(−0.31;1.0)	0.68(−0.74;4.4)	−0.08(−0.52;0.60)

Excess relative risk and 95% confidence intervals. In the restricted incidence analyses, time since median exposure has been restricted to more than 30 years for males and more than 35 years for females. Results for female mortality have large uncertainties and are not shown.

## Discussion

### Comparison to previous study

Compared to previous analysis [12], a substantial number of changes has been introduced. With regard to the data, the main differences are the use of the new dosimetry system MWDS-2008 and restriction to atherosclerotic induced deaths. Another methodology of analysis has been adopted: By performing an individual analysis, no information is lost in choosing Poisson groups. Compared to the stratified baseline applied in [12], the analytical modeling yields a more realistic, continuous baseline function, still being more parsimonious in the number of parameters. In addition, all available significant risk factors have been accounted for simultaneously. Furthermore, a non negligible part of the person years has been excluded: First, because preliminary investigations have uncovered a spurious feature in the calendar year dependence, the first years in the incidence analysis have been disregarded. Secondly, in order to avoid bias in the analysis on internal doses, person years before the first ascertainment of internal radiation burden have been excluded. On the other hand, reactor workers have been added to this analysis assuming no internal radiation burden. Finally, the analysis of response to external doses has been performed separately for males and females.

Despite these differences, results of the ERR per dose compare very well with [12]. Comparison can be performed directly for the unmodified LNT analysis with zero lag-time (table 2) as this analysis was also done separately for males and females in [12], tables 1, 2. It can be inferred that the best estimates have practically not changed but the ERR per dose became borderline significant for male mortality. Our results on internal doses, Table S1, should be compared with the analysis in [12], tables 3, 4, in which risk was adjusted for external radiation. For mortality, again the best estimate is not affected much due to the changes of the present analysis. But due to the exclusion of person years, incidence risk has formally lost precision and the best estimate has changed slightly.

**Table 4:** Cases and person years by categories of cumulative external doses.

Dose [Gy]	Incidence				Mortality	
	M		F		M	
	cases	person years	cases	person years	cases	person years
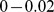	253	26416	238	15572	185	68933
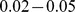	170	17233	115	6411	132	43086
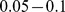	230	20076	160	7772	164	49846
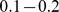	430	28318	247	10680	248	66589
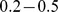	861	48120	342	13840	411	112170
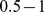	736	28789	254	11253	350	77167
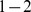	777	22060	252	9668	358	65443
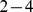	415	9883	111	3464	223	30210
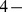	16	424	2	67	12	2702

### Comparison to other cohorts

Before comparing the risk estimates obtained in this study with other results of the literature we want to stress the multifactorial etiology of atherosclerosis [36]. Risk of cardiovascular diseases is known to vary greatly between different populations [37] and effectiveness of risk factors may vary between cohorts [38]. In addition, members of the different cohorts have been exposed to radiation in very different ways. Therefore, perfect agreement of the results from different cohorts cannot be expected.

Nevertheless, the meta-study [6] did not reveal significant heterogeneity in IHD radiation risk amongst different cohorts. Particularly, the risk derived from the Mayak Workers Cohort was found to be consistent to all other results – a fact that remains valid with this updated analysis. The most stringent other bounds on IHD risk are obtained from the cohort of atomic bomb survivors. A gender averaged excess relative risk at 1 Gy of 0.05 (−0.05; 0.16) was obtained for incidence when adjusting for smoking and drinking [39]; the mortality analysis yielded 0.02 (−0.10; 0.15) [40]. No analysis of lag-time was performed in either of these studies but we find consistency with either gender and any lag-time below 30 years, anyway, cf. table 2.

### Strengths and limitations

Before discussing further results, we want to acknowledge the strengths and limitations of this study.

First of all, losses of follow up are rare at least for residents of Ozyorsk [28]. Thus, selection bias should be small. Secondly, dose estimates are based on measurements. Therefore, although the error is rather large in the case of internal alpha exposures[31], it can be expected to be non-differential. Furthermore, regular medical surveillance is achieved due to annual examinations in the polyclinic for all employees and former employees still living in Ozyorsk. On the other hand, some differential bias could have been introduced due to additional examinations at SUBI, which have been performed more often for workers with jobs classified as harmful. This, however, cannot impact the result substantially as only a small part of the first incidence cases has been detected at SUBI. As the very long follow up inevitably implies some changes in the quality of surveillance, e.g. due to new technical capabilities, verification of the correct coding of the disease was performed using the original clinical data [28]. In summary, the good medical surveillance can be regarded as another strength of the Mayak Workers Cohort.

As already mentioned, many factors affect cardiovascular risk and proper adjustment of confounding is thus very important. In this study, we adjust for several individual risk factors directly and, in addition, the calendar year and birth year dependencies reflect changes in the average lifestyle. Moreover, we allow for work place dependence of cardiovascular risk by adjustment for work plant and date of first employment. Despite the extensive inclusion of covariables, still residual confounding cannot be excluded neither for the absolute value of the excess risk nor its progression in time. In particular, adjustment of job position may be crucial: For some exemplary group of workers at the Plutonium production plant, health of the cardiovascular system has been proven to depend on the job position [41]. In that study, the harm could be traced back to emotional stress but other very common labour conditions such as lack of exercise or shift work may also be important in that context. This can be problematic as dose may also be related to job position. We will come back to this issue in the following.

### Dose-response relationship

For male incidence, which comprises the analysis with the largest number of cases, and focusing on the late, main detrimental effects, the best fit is obtained with a step function. However, the LNT model cannot be excluded, cf. fig. 4A. A detailed comparison of different dose-response relationships has also been performed in [17] for the atomic bomb survivors for mortality from heart diseases. In contrast to our results, they found the LNT model to yield lowest 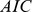 while a step model contributed only subordinate. Furthermore, the step was found at a substantially larger dose compared to our results. Therefore, the question on the shape of the dose response remains open.

Moreover, other analyses in this work show the existence of a peak in risk at about 20 to 200 mGy, cf. fig. 4B. The existence of non-linear effects at low doses is not new, cf. e.g. the mouse model [21], [22]. However, the observed peak contradicts the risk estimates derived from the cohort of Wismut miners [42]. There is also no such peak in the dose response for heart disease in the cohort of atomic bomb survivors [40] – a cohort, however, with a very different exposure history.

The peak could easily be induced by confounding: As already mentioned, it was noticed in [41] that work at certain job positions at Mayak PA affects cardiovascular health directly. In coincidence with the observed peak, workers with harmful labor conditions were found to be those with small but non-negligible exposure. Therefore, we do not consider this peak to be meaningful for radiation risk estimates but a better understanding of its nature is certainly advisable.

### Risk modification

Dependence of risk on time since exposure is a key ingredient of this study. In particular, we have demonstrated that main detrimental health effects in incidence occur more than 30 years after exposure. In comparison with the usually performed analysis with a lag-time of 10 years, the time since exposure effect is highly significant (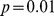 for males and 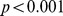 for females). We have illustrated in detail that the relevant time scale is indeed time since exposure and not e.g. the age of the individuals. It became also apparent that a step in risk more than 30 years after exposure describes the data much better compared to a slow increase over several decades. To show this, we have modified the ERR with time since median exposure – an approach allowing for more flexible analyses compared to the more common time-lagging of doses. However, in the case of the Mayak Workers Cohort where external exposures are often concentrated only on a time a span of a few years and under the assumption of an LNT dose-response, a step in time since median exposure yields similar results as utilization of a lag-time.

For female incidence, the time span from median exposure to the onset of main detrimental effects is longer compared to males. This observation goes with the fact that in general IHD develops later in life for females [43]. Another gender difference is the existence of a temporary protective effect before detrimental effects take over, cf. e.g. fig. 3. The existence of beneficial effects of low-dose radiation is well-known. For example, in clinical treatment of benign diseases, moderate doses of ionizing radiation have been shown to remedy inflammation and have been studied in vitro and in vivo [25]. However, the existence of such a strong gender difference in the excess relative risk is implausible even though there are significant differences in the baseline. In general, results from the male cohort may be more reliable not only as it includes more cases: Almost half of the women in the cohort have been employed prior to 1954, i.e. in the early years of operation where occupational health and safety was less developed. In contrast, less than a quarter of males in the cohort was hired during this time.

A priori, one might assume the probability of fatality of any disease not to depend strongly on whether this disease has been induced by radiation. As a consequence, the mortality ERR should be similar to the incidence ERR. This rationale, however, might be invalidated for example if radiation predisposes to a particular phenotype of atherosclerotic plaques [44]. Therefore, it is important to assess mortality independently. Indeed, no increase of risk with long times since exposure could be seen for mortality but results are not incompatible with incidence either: Comparing incidence with mortality the typical time for progression from first incidence to death needs to be considered. Within the 1443 persons in the cohort whose first IHD incidence and death from IHD occurred during the follow up, the mean time span between first incidence and death is 11 (5% and 95% percentiles: 0; 35) years. Therefore, mortality is not only expected to occur later in life but, due to the large variation in the time span from incidence to death, it is also plausible that any step-like feature in the time dependence of first incidences is diluted in mortality risk.

On the other hand, a strong increase in risk of IHD mortality with lag-time was found for the Techa River Cohort [45]. In addition, non-significant increase in risk with time since exposure has also been observed in [42] and for all circulatory diseases in [46]. Latency was also found to be important in ApoE deficient mice: The authors of [24] conclude that the heart can cope for a long time with structural and molecular changes after low radiation exposure until a breaking point occurs. Thus, a very delayed onset of IHD risk after low radiation exposure is consistent with other findings in the literature.

Note that on the contrary, a quite different result has been observed for persons who underwent radiotherapy thus being exposed locally to high doses and dose rates. For example, already 5 years after radiotherapy of breast cancer, frequency of major coronary events were significantly associated to dose [2]. In the US peptic ulcer cohort, even a decrease in risk of IHD mortality with increasing time since exposure is evident [47].

Finally, the late occurrence of first incidences needs to be discussed in the light of possible confounding. At first glance the observed time since exposure effect seems to coincide with the duration of a typical working life, i.e. first incidences seem to be raised after retirement. However, this hypothesis does not fit to the observed gender difference in the time span from exposure to incidence: On average, females have been exposed later in life and retire earlier. But the observed time span is longer for females. Moreover, an analysis with a baseline step function of time since first employment clearly showed that the onset of main detrimental effects is related to dose. Still, as explained above, a relation to dose might in principle emerge from confounding due to job position because the latter is related to dose. However, the analysis of time since exposure was based on the assumption of an LNT dose-response relationship and is therefore mostly sensitive to workers with high doses. These workers also drive the LNT analysis without modification, which showed good consistency with e.g. the atomic bomb survivors. In [41], a group of workers with low doses had been found to have harmful labor conditions. In an LNT-based analysis, however, they have comparably lower impact. We conclude that confounding due to job position is unlikely for this specific case.

After analysis of risk progression, we have analyzed radiation risk modification by other risk factors. Very interestingly, we observe body mass at the preemployment examination to be related to radiation risk: In male incidence, underweight workers show significantly higher excess relative risk compared to persons with normal weight, cf. Table S2. Although a causal relation may be possible, it is again difficult to exclude confounding. It might also be important to note that 50% of the workers hired with underweight have been employed before 1954, i.e. in the post war years, and some gained body mass very rapidly after employment. Hence, a comparison to other studies would be very valuable but we are not aware of any other epidemiological study on this issue.

## Conclusions

The Mayak Workers Cohort has enough statistical power not only to observe radiation effects in ischemic heart diseases but also to look at gender differences, dose modifications and the shape of the dose response. As any occupational cohort, however, it faces the problems that persons are selected into specific job positions and that work may also have a direct health impact, specific to the position. These problems are particularly severe for atherosclerosis, which is known to depend strongly on various lifestyle factors. Therefore, confounding plays an important role and much effort has been spent to detect possible confounders and adjust for them.

Still, especially in the low dose regime the analysis of the shape of the dose response is likely to be distorted by residual confounding. When applying the LNT model, however, the fit is mostly sensitive to workers with higher doses and the impact of confounding should be minor. Indeed, our results are in good agreement with other studies on ischemic heart disease. For example, the results are consistent within the error bars with the atomic bomb survivor data [39], [40]; the latter are, however, insignificant when focussing on ischemic heart disease only. Compared to the LNT model, we found a step function to be slightly favored. However, it remains speculative to draw any conclusion about the dose-response relationship.

We analyzed in detail the dependence of incidence risk on age, age at exposure, time since exposure and duration of half exposure. Our main finding is that main detrimental effects occur very late, more than about 30 years after median exposure. This result is statistically strongly supported and stable against some tests for possible confounding.

## Supporting Information

Appendix S1 **Details on the baseline and the models for the dose-response relationship.**
(PDF)

Table S1
**Excess relative risk per dose [** 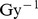 **] for internal radiation for various lag-times.**
(PDF)

Table S2 **Parameters of modification of the external dose response by other risk factors.**
(PDF)
