# Supplementary material for: Ischemic Heart Disease in Workers at Mayak PA: Latency of Incidence Risk after Radiation Exposure
Source: PLoS One. 2014 May 14;9(5):e96309. doi: 10.1371/journal.pone.0096309 (PMC4020749; doi:10.1371/journal.pone.0096309)
Supplement: Appendix S1 — Details on the baseline and the models for the dose-response relationship. (PDF) [file pone.0096309.s001.pdf]

A Appendix

A.1 Notation

To keep the tables in the supplementary material compact, estimates with a maximum likelihood estimation θ and a likelihood interval of $[\theta - \sigma_1, \theta + \sigma_2]$ are written as $\theta^{+\sigma_1}_{-\sigma_2}$.

A.2 The Baseline

A.2.1 The baseline template

The baseline is parameterized by the following template:

$$\begin{aligned} h_0 &= e^{\psi_{\text{cat}}+\psi_{\text{time}}+\psi_{\text{emigration}}} \\ \psi_{\text{cat}} &= \psi_{\text{smoking}} + \psi_{\text{drinking}} + \psi_{\text{bmi}} + \psi_{\text{blood pressure}} + \psi_{\text{plant}} \\ \psi_{\text{time}} &= \psi_{\text{age}} + \psi_{\text{birth}} + \psi_{\text{calendar}} + \psi_{\text{employment}} \end{aligned} \tag{A1}$$

Summands in ψ_{cat} evaluate to zero for non-smoker, non-drinker, for persons with normal body mass index, normal blood pressure and for workers at the reactors. Otherwise they evaluate to some value determined by the fit. The time dependent functions are defined as:

$$\begin{aligned} \psi_{\text{age}} &= \psi_0 + \psi_1 \log\frac{a}{60} + \psi_2 \log^2\frac{a}{60} + \sum_i \alpha_i \log^2\frac{a}{\vartheta_{\alpha,i}} \cdot \Theta(a - \vartheta_{\alpha,i}) \\ \psi_{\text{birth}} &= \beta_1 \frac{b-1900}{10} + \beta_2 \frac{(b-1900)^2}{100} \\ \psi_{\text{calendar}} &= \sum_i \gamma_i \frac{\text{LT}(b+a-\vartheta_{\gamma,i})}{10} \\ \psi_{\text{employment}} &= \delta_1 \frac{f-1950}{10} + \delta_2 \frac{(f-1950)^2}{100} \\ \psi_{\text{emigration}} &= \epsilon \cdot \Theta(b+a-m) \end{aligned} \tag{A2}$$

Here, a, b, f and m are abbreviations for age, birth date, date of first employment, and date of emigration from Ozyorsk, respectively. Lowercase Greek symbols denote fit parameters. We have introduced the Heaviside step function Θ and a function $\text{LT}(t)$:

$$\Theta(t) = \begin{cases} 0 & \text{for } t < 0 \\ 1 & \text{for } t \geq 0 \end{cases} \qquad \text{LT}(t) = \begin{cases} 0 & \text{for } t < 0 \\ t & \text{for } t \geq 0 \end{cases} \tag{A3}$$

Depending on the endpoint, radiation type and gender under consideration, only a subset of the parameters introduced in this template significantly deviates from zero. In the analyses of the dose response, only significant parameters are to be maintained. These are found by iterative testing. In doing so, the dose response is parameterized by an excess relative risk model:

$$h = h_0(1 + ERR_{\text{ext}})(1 + ERR_{\text{int}}) \tag{A4}$$

where an LNT dose-response relationship is applied to ERR_{ext} with a lag-time of 10 years. In order to be able to use all persons and person years available for the analysis of external doses, we abandon to correct for internal doses. This approach will be justified a posteriori by the absence of a significant dose response for internal doses. For the determination of the baseline model related to the analysis of internal doses, the LNT model is applied to ERR_{ext} as well as ERR_{int}.

For convenience, we define here also the excess absolute risk (EAR) model:

$$h = h_0 + EAR_{\text{ext}} + EAR_{\text{int}} \tag{A5}$$

Attribute	Category	Incidence		Mortality	
		M	F	M	F
ψ_{smoking}	smoker	$\mathbf{0.15}^{+0.09}_{-0.08}$	$0.04^{+0.22}_{-0.23}$	$\mathbf{0.58}^{+0.13}_{-0.12}$	$\mathbf{0.99}^{+0.34}_{-0.37}$
ψ_{drinking}	drinker	$-0.02^{+0.21}_{-0.20}$	$-0.01^{+0.10}_{-0.10}$	$0.16^{+0.25}_{-0.23}$	$\mathbf{-0.31}^{+0.22}_{-0.23}$
ψ_{bmi}	< 18.5 kg/m^2	$0.03^{+0.32}_{-0.35}$	$0.18^{+0.48}_{-0.57}$	$0.30^{+0.42}_{-0.49}$	$-0.24^{+0.98}_{-1.43}$
	≥ 25 kg/m^2	$\mathbf{0.17}^{+0.09}_{-0.09}$	$\mathbf{0.17}^{+0.12}_{-0.12}$	$\mathbf{0.24}^{+0.13}_{-0.13}$	$0.20^{+0.23}_{-0.23}$
$\psi_{\text{blood pressure}}$	$> 140/90$ mmHg	$\mathbf{0.16}^{+0.09}_{-0.09}$	$0.04^{+0.15}_{-0.16}$	$\mathbf{0.28}^{+0.11}_{-0.12}$	$\mathbf{0.40}^{+0.24}_{-0.26}$
ψ_{plant}	radiochemical	$\mathbf{-0.08}^{+0.07}_{-0.07}$	$0.04^{+0.15}_{-0.14}$	$-0.01^{+0.10}_{-0.10}$	$-0.06^{+0.26}_{-0.26}$
	plutonium	$0.04^{+0.09}_{-0.09}$	$\mathbf{0.13}^{+0.11}_{-0.11}$	$0.04^{+0.10}_{-0.10}$	$\mathbf{-0.21}^{+0.20}_{-0.21}$

Table A1. Baseline parameters associated with categorical data and their 95% confidence intervals. The parameters for non-smoker, non-drinker, normal body mass index, normal blood pressure and work at the reactors are defined to be zero and are not shown. The parameters corresponding to unknown information are not shown either. Significant parameters are marked bold. In the related analyses, external doses were accounted for by applying an LNT model.

A.2.2 Non-radiation risk factors

The results for the categorical baseline parameters are presented in table A1 for the analysis of external doses. Only parameters significantly deviating from zero are used in the analyses. An exception are the parameters related to the categories of missing information. Those were kept as free parameters if the corresponding attribute contains a significant category. Likewise values and confidence intervals have been derived in fixing to zero all non-significant parameters but the variable under question. Results from the cohort on internal doses are not shown but are similar.

The established risk factors of smoking, hypertension and overweight could be confirmed. Interestingly, they seem to have stronger impact for mortality compared to incidence. Drinking was significantly associated with risk only for female mortality, where a protective effect could be seen. Lastly, work plant is a significant covariable for some analyses but the trend is unclear. Significance of work plant disappears when taking into account internal doses and restricting the cohort accordingly.

The other parameters of the baseline for analysis of external doses are shown in table A2. Again, results for the cohort on internal doses are not shown. As pointed out already in the introduction, the calendar year dependence of the hazard exhibits kinks both for incidence and mortality. Concerning age dependence, it is interesting to note that at high ages the incidence hazard levels off, while this could not be observed for mortality. The precise trends with calendar year, age and birth year should be interpreted with some caution as separation of age, birth year and calendar year is difficult. As expected, risk is highest for workers hired in the early years of operation. The apparent risk of emigrated males is about 0.9 times the risk of inhabitants and 0.8 for emigrated females, cf. the methods section for possible reasons.

Attribute	Var.	Incidence		Mortality	
		M	F	M	F
ψ_{age}	ψ_0	15.2	9.2	5.6	5.4
	ψ_1	27.1	-0.3	5.9	8.2
	ψ_2	14.3	-13.8		
	α_1	-204.5			
	α_2	184.6			
	$\vartheta_{\alpha,1}$	41.0			
	$\vartheta_{\alpha,2}$	42.0			
ψ_{birth}	β_1	0.15	0.02	0.01	
	β_2	-0.05	-0.17	0.04	
ψ_{calendar}	γ_1	-0.8	1.5	1.2	
	γ_2	6.2	-2.1	-1.7	
	γ_3	-5.2	12.9		
	γ_4	-2.1	-16.5		
	γ_5		5.4		
	$\vartheta_{\gamma,1}$	1981.0	1976.9	1991.5	
	$\vartheta_{\gamma,2}$	1992.1	1981.4	1993.8	
	$\vartheta_{\gamma,3}$	1993.1	1992.1		
	$\vartheta_{\gamma,4}$	2003.5	1993.1		
	$\vartheta_{\gamma,5}$		1994.3		
$\psi_{\text{employment}}$	δ_1	-0.28	-0.14		
	δ_2	0.10			
$\psi_{\text{emigration}}$	ϵ			-0.11	-0.26

Table A2. Baseline parameters not associated to categorical data. Parameters that do not significantly deviate from zero, are kept empty in the table. In the related analyses, external doses were accounted for by applying an LNT model.

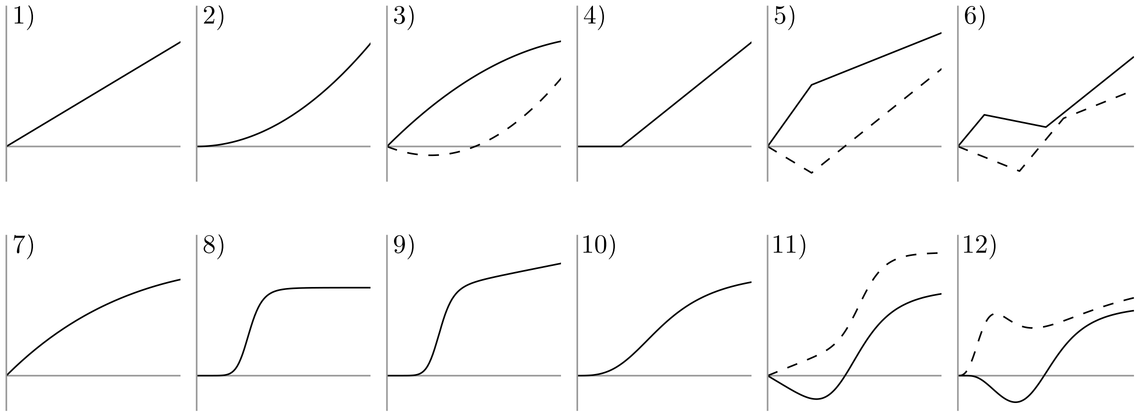

Figure A1. Typical shapes of the functions tested for as dose response. Additional dashed lines have been plotted to show the flexibility of some of the functions. The functions are called 1) LNT 2) Quadratic 3) Linear-quadratic 4) Linear-threshold 5) Two-line-spline 6) Three-line-spline 7) Linear-exponential 8) Step 9) Step-linear 10) Sigmoid 11) Hormesis I 12) Hormesis II

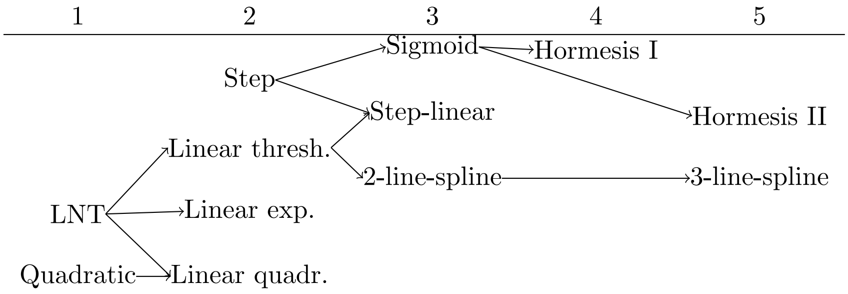

Figure A2. Number of parameters of the studied dose response models and their relations. Two models are nested if they are connected by successive arrows.

A.3 Functions of the dose response

The shape of the dose response is analyzed with the following twelve functions, which are sketched in fig. A1.

$$\begin{aligned}
&\text{1) LNT:} && ERR(d) = \lambda\, d \qquad \text{(A6)}\\
&\text{2) Quadratic:} && ERR(d) = \lambda\, d^2\\
&\text{3) Linear-quadratic:} && ERR(d) = \lambda_1\, d + \lambda_2\, d^2\\
&\text{4) Linear-threshold:} && ERR(d) = \lambda\ \mathrm{LT}(d-\vartheta)\\
&\text{5) Two-line-spline:} && ERR(d) = \lambda_0\, d + \lambda_1\ \mathrm{LT}(d-\vartheta)\\
&\text{6) Three-line-spline:} && ERR(d) = \lambda_0\, d + \lambda_1\ \mathrm{LT}(d-\vartheta_1) + \lambda_2\ \mathrm{LT}(d-\vartheta_2)\\
&\text{7) Linear-exponential:} && ERR(d) = \lambda_1\, d\cdot\exp(-\lambda_2\, d)\\
&\text{8) Step:} && ERR(d) = \lambda_0 - \frac{\lambda_0}{1+\left(\frac{d}{\vartheta}\right)^8}\\
&\text{9) Step-linear:} && ERR(d) = \lambda_0 - \frac{\lambda_0}{1+\left(\frac{d}{\vartheta}\right)^8} + \lambda_2\ \mathrm{LT}(d-\vartheta)\\
&\text{10) Sigmoid:} && ERR(d) = \lambda_0 - \frac{\lambda_0}{1+\left(\frac{d}{\vartheta}\right)^{\lambda_1}}\\
&\text{11) Hormesis I:} && ERR(d) = \lambda_0 - \frac{\lambda_0 + \lambda_2\, d}{1+\left(\frac{d}{\vartheta}\right)^{\lambda_1}}\\
&\text{12) Hormesis II:} && ERR(d) = \lambda_0 - \frac{\lambda_0 + \lambda_2 \exp\left(-\frac{\vartheta_2}{d}\right)}{1+\left(\frac{d}{\vartheta_1}\right)^{\lambda_1}}
\end{aligned}$$

Here, we have used the function LT, cf. eq. (A3). The hormesis I function was introduced in [1], hormesis II is based on [2] but has been complemented by an additional parameter to get rid of the dependence on the choice of units.

Instead of an instantaneous step we use a smooth step function. The slope of this step function is set by the choice $\lambda_1 = 8$, which approximately resembles the smoothing effect of dose uncertainty assuming dose uncertainty to follow a lognormal distribution with parameter $\sigma = \log(1.26)$ [3]. Very steep steps typically yield a lower deviance, a fact that can be understood as follows. In a model with an instantaneous step, person years are partitioned into years of baseline risk and of elevated risk. If

the position of the step is incremented, baseline risk is attributed to more person years. Therefore, the deviance improves with an increment of the position of the step as long as the number of cases under elevated risk is not affected. However, if the position of the step passes a dose of a case, the deviance exhibits a jump. If the position approaches the dose of a case from the left, the deviance is in a minimum. The more persons are included in the cohort, the smaller are the gaps between doses of cases and the minima become very narrow. When using a smooth step function with a gradient covering several doses of cases, these narrow minima and maxima are averaged out.

References

[1] Brain P, Cousens R (1989) An equation to describe dose responses where there is stimulation of growth at low doses. Weed Research 29: 93–96.

[2] Cedergreen N, Ritz C, Streibig JC (2005) Improved empirical models describing hormesis. Environ Toxicol Chem 24: 3166–72.

[3] Vasilenko EK, Khokhryakov VF, Miller SC, Fix JJ, Eckerman K, et al. (2007) Mayak worker dosimetry study: an overview. Health Phys 93: 190–206.
