# Supplementary material for: Ischemic Heart Disease in Workers at Mayak PA: Latency of Incidence Risk after Radiation Exposure
Source: PLoS One. 2014 May 14;9(5):e96309. doi: 10.1371/journal.pone.0096309 (PMC4020749; doi:10.1371/journal.pone.0096309)
Supplement: Table S1 — Excess relative risk per dose [ ] for internal radiation for various lag-times. (PDF) [file pone.0096309.s002.pdf]

Lag-time	0 years	10 years	20 years	30 years
Incidence	-0.01(-0.05;0.05)	-0.02(-0.07;0.09)	-0.03(-0.10;0.17)	-0.16(-0.16;0.09)
Mortality	0.07(-0.03;0.20)	0.08(-0.03;0.26)	0.13(-0.04;0.38)	0.27(-0.03;0.74)

Table S1. Excess relative risk per dose [Gy^{-1}] **for internal radiation with 95% confidence levels for various lag-times.** To correct for external radiation, an LNT model with the same lag-time has been applied. The confidence intervals for incidence are bound from below by the requirement of a positive hazard, $ERR_{\mathrm{int}} > -1$, for the worker with highest dose.
