# Supplementary material for: Ischemic Heart Disease in Workers at Mayak PA: Latency of Incidence Risk after Radiation Exposure
Source: PLoS One. 2014 May 14;9(5):e96309. doi: 10.1371/journal.pone.0096309 (PMC4020749; doi:10.1371/journal.pone.0096309)
Supplement: Table S2 — Parameters of modification of the external dose response by other risk factors. (PDF) [file pone.0096309.s003.pdf]

Attribute	Category	Incidence	
		M	F
$\psi^{\text{mod}}_{\text{smoking}}$	smoker	$0.2^{+\infty}_{-1.0}$	$0.1^{+1.3}_{-\infty}$
$\psi^{\text{mod}}_{\text{drinking}}$	drinker	$4.1^{+\infty}_{-5.2}$	$-0.1^{+0.9}_{-1.3}$
$\psi^{\text{mod}}_{\text{bmi}}$	< 18.5 kg/m^2	$\mathbf{1.9}^{+1.1}_{-1.7}$	$2.4^{+1.4}_{-3.0}$
	≥ 25 kg/m^2	$-0.2^{+1.2}_{-\infty}$	$-1.1^{+1.2}_{-\infty}$
$\psi^{\text{mod}}_{\text{blood pressure}}$	$> 140/90$ mmHg	$-0.7^{+1.1}_{-\infty}$	$-0.8^{+1.3}_{-\infty}$

Table S2. Parameters of modification of the external dose response by other risk factors and their 95% confidence intervals. Analysis is restricted to doses for which median exposure is more than 30 years ago for males (and more than 35 years for females), and is based on an LNT model. Ratios of the excess relative risk for persons with a specific risk factor to persons without may be calculated as $\exp(\psi^{\text{mod}}_{\text{cat}})$.
